# A genetic toolbox to empower *Paracoccus pantotrophus* DSM 2944 as a metabolically versatile SynBio chassis

**DOI:** 10.1186/s12934-024-02325-0

**Published:** 2024-02-15

**Authors:** Upasana Pal, Denise Bachmann, Chiara Pelzer, Julia Christiansen, Lars M. Blank, Till Tiso

**Affiliations:** 1 Institute of Applied Microbiology, RWTH Aachen University, Aachen, Germany; 2 Chair of Microbiology, Technical University of Munich, Freising, Germany

**Keywords:** *Paracoccus*, Genetic toolbox, SynBio chassis, Adaptive laboratory evolution, Plastics, Bioeconomy, Metabolic engineering

## Abstract

**Background:**

To contribute to the discovery of new microbial strains with metabolic and physiological robustness and develop them into successful chasses, *Paracoccus pantotrophus* DSM 2944, a Gram-negative bacterium from the phylum Alphaproteobacteria and the family Rhodobacteraceae, was chosen. The strain possesses an innate ability to tolerate high salt concentrations. It utilizes diverse substrates, including cheap and renewable feedstocks, such as C1 and C2 compounds. Also, it can consume short-chain alkanes, predominately found in hydrocarbon-rich environments, making it a potential bioremediation agent. The demonstrated metabolic versatility, coupled with the synthesis of the biodegradable polymer polyhydroxyalkanoate, positions this microbial strain as a noteworthy candidate for advancing the principles of a circular bioeconomy.

**Results:**

The study aims to follow the chassis roadmap, as depicted by Calero and Nikel, and de Lorenzo, to transform wild-type *P. pantotrophus* DSM 2944 into a proficient SynBio (Synthetic Biology) chassis. The initial findings highlight the antibiotic resistance profile of this prospective SynBio chassis. Subsequently, the best origin of replication (ori) was identified as RK2. In contrast, the non-replicative ori R6K was selected for the development of a suicide plasmid necessary for genome integration or gene deletion. Moreover, when assessing the most effective method for gene transfer, it was observed that conjugation had superior efficiency compared to electroporation, while transformation by heat shock was ineffective. Robust host fitness was demonstrated by stable plasmid maintenance, while standardized gene expression using an array of synthetic promoters could be shown. pEMG-based scarless gene deletion was successfully adapted, allowing gene deletion and integration. The successful integration of a gene cassette for terephthalic acid degradation is showcased. The resulting strain can grow on both monomers of polyethylene terephthalate (PET), with an increased growth rate achieved through adaptive laboratory evolution.

**Conclusion:**

The chassis roadmap for the development of *P. pantotrophus* DSM 2944 into a proficient SynBio chassis was implemented. The presented genetic toolkit allows genome editing and therewith the possibility to exploit *Paracoccus* for a myriad of applications.

## Background

The concept of a microbial chassis captures the true sense of a genetically engineered microbe possessing genetically, and metabolically relevant properties to serve functions in the light of applied microbiology. The term chassis started gaining popularity in the early 2000s when microbiologists engineered genetically modified strains for à *la carte* applications [1]. Although there are several widely used microbial chassis organisms currently in use, *e.g.*, *Escherichia coli* as the flagship bacterium [2], *Bacillus subtilis* as a robust workhorse for heterologous protein production [3, 4], *Saccharomyces cerevisiae* for bioethanol production [5, 6], and many more [7], the search for new and improved microbial hosts is far from being over. It has been long since argued that a handful of microbes, although having immense potential and being amenable to genetic modification, cannot be the solution to every challenge encountered in using microbes in a circular bioeconomy. There is a clear need to establish further chassis organisms with robust physiology (*e.g.*, against temperature and osmotic stresses) and versatile metabolism [8], including the ability to harness cost-effective and sustainable resources while concurrently facilitating the synthesis of bio-based products [9]. With the help of Cultivarium (www.cultivarium.org) and other initiatives, technological improvements to access previously untouched microbes become more accessible.

**Fig. 1:**
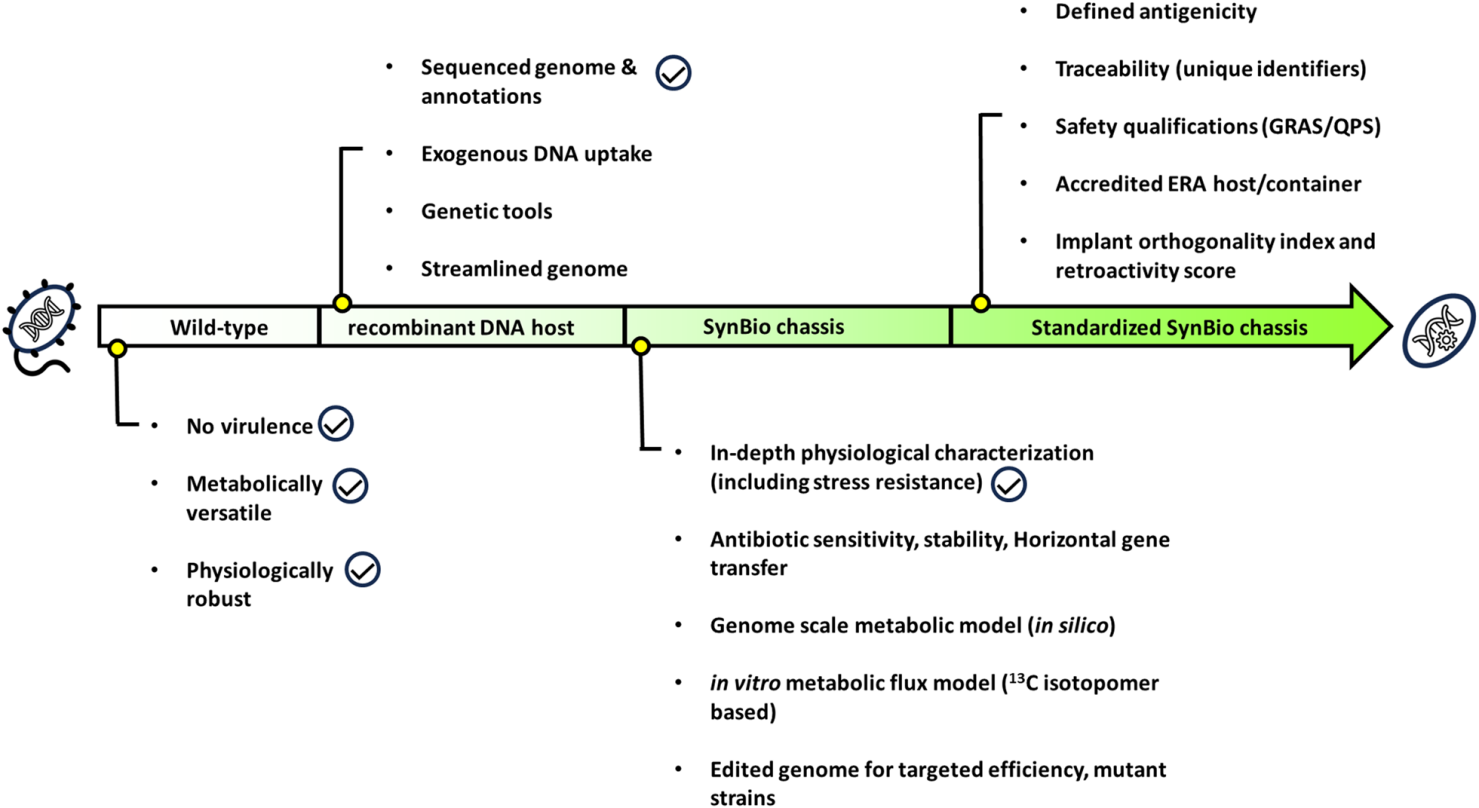
Roadmap highlighting the development of a SynBio chassis. The figure is derived from the proposals of Calero and Nikel [7] and de Lorenzo [1], with the latter suggesting a defined nomenclature (wild-type, recombinant DNA host, SynBio chassis, and standardized SynBio chassis) aiding in regulatory guidelines. The check marks shown in the figure signify milestones that have already been reached for *P. pantotrophus* DSM 2944 in previous studies [19, 24]

Based on the above-mentioned arguments, Calero and Nikel described six major laboratory research steps required to develop wild-type bacteria into a chassis [7]. These milestones include (1) genome sequencing with high-quality annotations, (2) development of a robust genetic toolbox (comprised of replicative and suicide plasmids, promoters, and other precise genetic engineering tools), (3) in silico metabolic model backend with experimental validation, (4) in-depth physiological characterization, (5) construction of genome reduced strains for increased biomass or product formation by curbing unnecessary reactions, and finally (6) a mutant strains with improved functions (as shown in Fig. 1) [7].

Furthermore, the concept of constructing a new chassis strain was advanced with the help of a more recent roadmap developed by de Lorenzo et al. [1]. This roadmap elucidated a step-by-step guide for constructing a novel chassis microorganism created with the aid of synthetic biology (SynBio), keeping in mind the industrial and regulatory acceptance guidelines [10]. Thus, de Lorenzo aptly coined the term SynBio chassis. Unlike Calero’s approach, this updated approach although comprising laboratory-based research goals, was further supported with a definite nomenclature signifying the development of the chassis from one stage to the next. The following four stages depict the promotion of an environmental isolate to a fully-fledged standardized SynBio chassis.

1. Wild type or isolate: selection of a non-virulent wild-type strain possessing metabolic or physiological advantage.
2. Recombinant DNA (rDNA) host: conversion of the isolate into a reusable biological platform organism, characterized by the absence of virulence genes, the ability of genetic modification, and the availability of a robust genetic toolbox.
3. SynBio chassis: the rDNA host is additionally characterized by high-quality genome sequencing, factors concerning stress tolerance, and defined energy metabolism. Also, information regarding antibiotic sensitivity and the capability of horizontal gene transfer (HGT) should be available, and finally, the genome should be edited for improved efficiency in targeted approaches [1].
4. Standardized SynBio chassis: finally, the engineered SynBio chassis undergoes comprehensive analysis, and traceability is established through the incorporation of unique gene identifiers. This is subsequently followed by attaining the designation of being generally recognized as safe (GRAS) status [11] and accompanied by a qualified presumption of safety (QPS) certification, which ultimately gets applied using the environmental risk assessment (ERA) norms [10]. These evaluations collectively render the strain eligible for expedited approval in industrial and food-based applications [1]. Additionally, this step is also reinforced in chassis development by Adams [12], with an additional characteristic of having a host-vector biosafety (HVB) certification [13] for a smooth transition of the laboratory-constructed SynBio chassis to varied industrial applications.

In 1983, *Paracoccus pantotrophus* DSM 2944 (then known as *Thiosphaera pantotropha*) was isolated from a denitrifying, sulfide-oxidizing effluent treatment plant located in Delft, The Netherlands, possessing the ability to grow aerobically and anaerobically on reduced sulfur compounds and hydrogen while fixing carbon dioxide [14–16], and tolerating short-chain alkanes as carbon source, promoting bioremediation [17]. Salient features also include efficient energy management due to the absence of flagella and related energy sinks [18] and the presence of a complete and extremely versatile electron-transport chain. Furthermore, out of over one hundred *Paracoccus* species, only one reported strain, *Paracoccus yeei* CCUG 32053, which is phylogenetically distant from our selected chassis strain, *P. pantotrophus* DSM 2944 [19], was declared to be an opportunistic human pathogen [20, 21]. All these features render *P. pantotrophus* DSM 2944 a promising candidate for exploration as a production host for industrial applications. Moreover, in-depth physiological characterization revealed the organism’s resistance against high salinity (> 10% NaCl) and good thermotolerance (up to 45°C), thereby promoting auto sterility (these extreme conditions are unsuitable for the growth of contaminating microbes) and economically feasible industrial applications [22]. *P. pantotrophus* also possesses a versatile metabolic arsenal, including the ability to utilize the C1 compound formate and C2 compound ethylene glycol (EG), organic acids, and alcohols, all coupled with the production of short-chained biopolymer, polyhydroxybutyrate [19, 23].

**Fig. 2:**
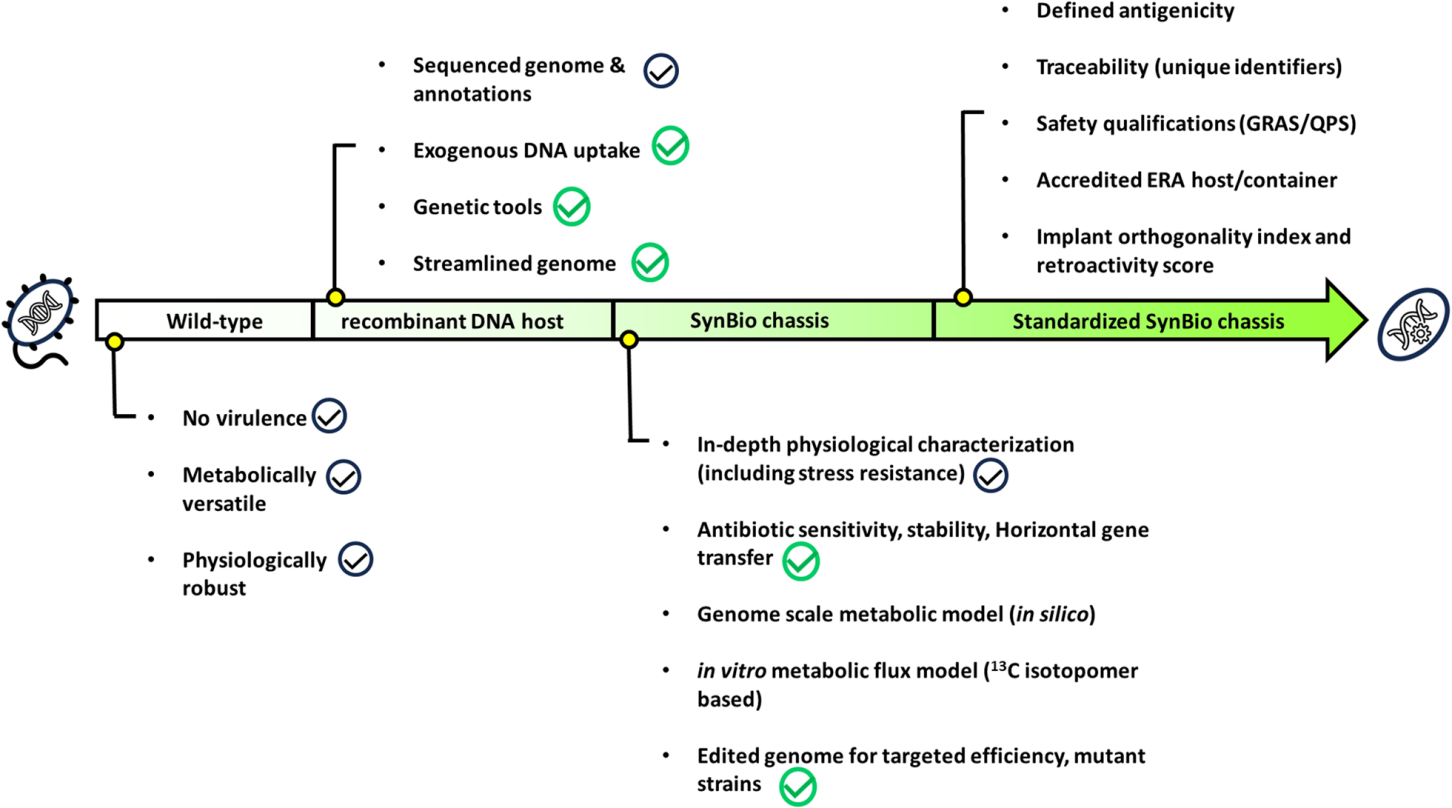
Completed milestones in this study. While the check marks in black represent the starting point of the development of *P. pantotrophus* as a chassis strain (see also Fig. 1), the check marks in green signify the advancements that have been made in this scientific quest for promoting *P. pantotrophus* DSM 2944 from a wild-type organism with interesting properties to a novel SynBio chassis

This study presents the development of *P. pantotrophus* DSM 2944 into an rDNA host. The genetic toolbox comprises now essential genetic tools (e.g., plasmids, including origins of replication, promoters, information on plasmid stability, antibiotic resistance profiling, and genome editing through gene deletion and integration). Importantly, the strain can be manipulated by exogenous DNA uptake through transformation and conjugation methods. Backed by previous studies reporting whole-genome sequencing and annotation [24] and in-depth studies of physiology and tolerance against abiotic stressors [19], and here presented adaptive laboratory evolution experiments, the newly developed rDNA host was further promoted following the chassis roadmap. The tools available are showcased to engineer *P. pantotrophus* DSM 2944 to grow on the PET monomers ethylene glycol and terephthalic acid at a high rate. In summary, *P. pantotrophus* DSM 2944 is developed into a SynBio chassis (Fig. 2), further paving the path to becoming a host for future industrial applications [7].

## Results

### Designing a suitable plasmid for efficient genetic engineering

To develop *P. pantotrophus* DSM 2944 into a SynBio chassis, a genetic engineering toolbox was constructed. Special focus was given to the curation of a replicative plasmid (including oris, promoters, selective, and counter-selective markers) and a non-replicative plasmid (which can be utilized for gene integration and deletion). Studies involving plasmid stability and DNA transfer techniques (transformation/conjugation) conclude the toolbox construction.

#### Antibiotic resistance profiling for suitable marker selection

**Fig. 3:**
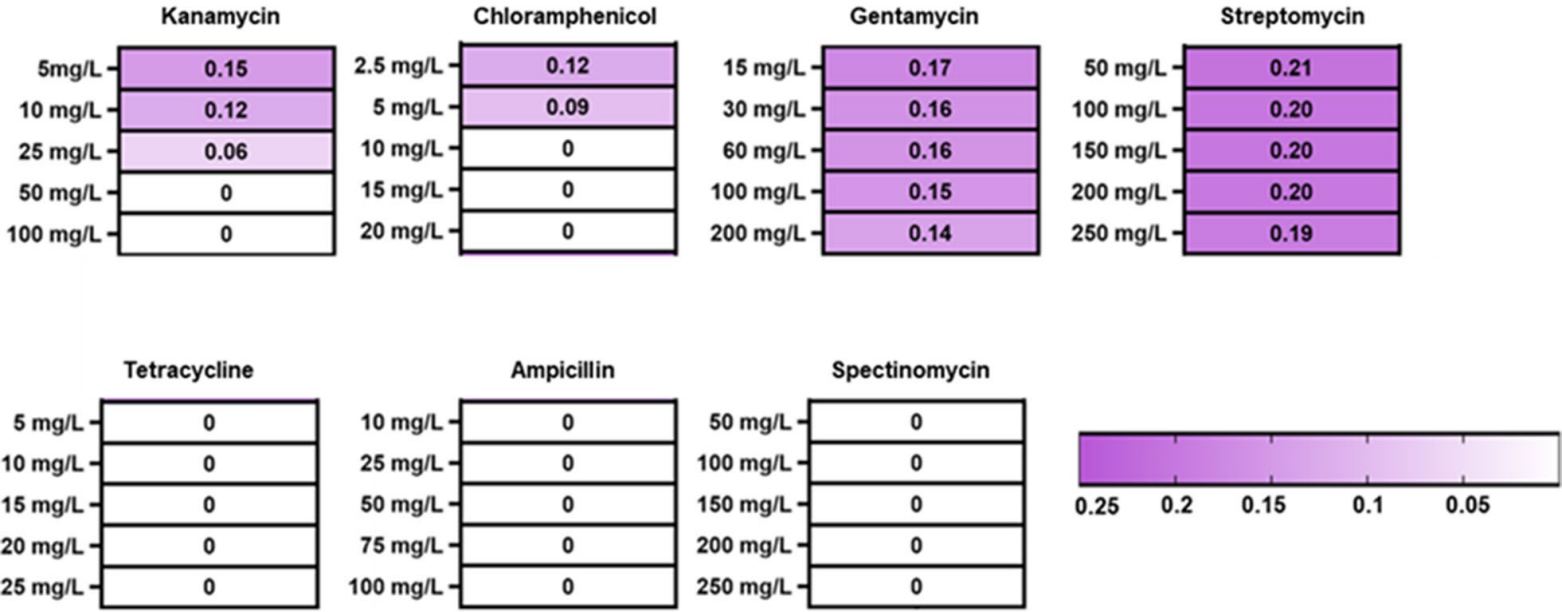
Antibiotic screening. The heatmap depicts the growth rate (h^−1^) of *P. pantotrophus* DSM 2944 towards seven different antibiotics with varying concentrations. The intensity of the color is directly proportional to higher growth. The experiment was performed in triplicates in 96-well plates with lysogeny broth (LB) and the respective antibiotic concentrations using the Growth Profiler

To determine the antibiotic resistance profile of *P. pantotrophus*, the cells were grown in the presence of seven different antibiotics with varying concentrations; namely kanamycin (5–100 mg/L), gentamycin (15–200 mg/L), ampicillin (10–100 mg/L), chloramphenicol (2.5–20 mg/L), streptomycin (50–250 mg/L), tetracycline (5–25 mg/L), and spectinomycin (50–250 mg/L). The range of the different antibiotics was selected to cover the standard concentrations as described by the Barrick Lab [25]. The minimum inhibitory concentration (MIC) for all antibiotics specific to *P. pantotrophus* is determined based on the resulting growth rates (Fig. 3).

*P. pantotrophus* has very high resistance to streptomycin and gentamycin (200 and 250 mg/L respectively), which makes these antibiotics suitable as selection markers, *e.g.*, during conjugation, against the growth of other microbes such as *E. coli*. On the other hand, at 25 mg/L and 5 mg/L for kanamycin and chloramphenicol, respectively, the MIC is reached. Finally, no growth was obtained at any ampicillin, tetracycline, and spectinomycin concentration, suggesting high sensitivities and rendering these antibiotics suitable reporter genes for gene transfer technologies. Thus, MICs of only two antibiotics (kanamycin and chloramphenicol) were determined. These findings regarding the specific antibiotic resistance profile of *P. pantotrophus* DSM 2944, aid in the one-step selection of transconjugants with kanamycin (50 mg/L) as selective and streptomycin (50 mg/L) as a counter-selective marker. With suitable antibiotics in hand, a plasmid could be developed.

#### Finding the best origin of replication

It is critical for a genetic toolbox to identify two types of replication origins. One that can replicate in *P. pantotrophus* DSM 2944 and another one that is incapable of replication and thus is suitable for application in a suicide vector.

Nine plasmids were selected from the Standard European Vector Architecture (SEVA) platform (http://seva.cnb.csic.es) [26] with similar backbones containing a multiple cloning site and a kanamycin resistance gene. The only difference between the different SEVA plasmids was the varying oris. The nine oris were R6K [27], RK2 [28], pBBR1 [29], pRO1600/ColE1 [26], RFS1010 [26], p15A [30], pSC101 [31], pUC [32], and pBR322/ROP [33].

The *P. pantotrophus* cells were treated to be electrically competent and a constant volume of 100 µL of these electrocompetent cells was then transformed with a fixed concentration of 100 ng of the above-mentioned SEVA plasmids using electroporation [34]. After two days, single colonies were observed on LB plates containing 50 mg/L kanamycin, which were counted to assess the functionality of the oris. Given the fixed quantities of plasmid, electrocompetent cells, and the antibiotic, the assumption was made that ori compatibility exhibited a direct proportionality to the number of transformed colonies observed.

The results (Table 1) show the low copy number, broad host range ori R2K [35] as the best replicative origin in *P. pantotrophus* DSM 2944 (possessing the highest number of transformants and was thus deemed as the most compatible ori). Whereas high copy-number plasmid oris pRO1600/ColE1, (in the case of *E. coli*) [36, 37] and RFS1010 (*Pseudomonas*) [38] when transformed resulted only in a few colonies, hinting that they possess low compatibility with *P. pantotrophus* DSM 2944 as the host. This can stem from the fact that higher copy number plasmids generally cause metabolic burden to the host [39]. No transformants were obtained with any of the other origins, thereby acting as potential oris for suicide vectors. Out of which, ori R6K was chosen as the basis for a suicide plasmid for future genome-based editing.

**Table 1:** Origin of replication (ori) compatibility.

ori	Name	Length of the plasmid (bp)	Genebank entry	Number of transformed colonies	Ori compatibility with *P. pantotrophus* DSM 2944
*R6K*	pSEVA211	1,993	JX560326	*No colonies*	*Non-compatible*
*RK2*	pSEVA221	3,823	JX560327	*1523*	*Best compatible*
*pBBR1*	pSEVA231	3,123	JX560328	*No colonies*	*Non-compatible*
**pRO1600/ColE1**	pSEVA241	3,570	JX560329	**7**	**Low compatibility**
**RFS1010**	pSEVA251	5,275	JX560330	**15**	**Low compatibility**
*p15A*	pSEVA261	2,333	pSEVA261.gbk	*No colonies*	*Non-compatible*
*pSC101*	pSEVA271	3,061	pSEVA271.gbk	*No colonies*	*Non-compatible*
*pUC*	pSEVA281	2,530	pSEVA281.gbk	*No colonies*	*Non-compatible*
*pBR322/ROP*	pSEVA291	2,986	pSEVA291.gbk	*No colonies*	*Non-compatible*

The table showcases the compatibility of different oris with *P. pantotrophus* DSM 2944. The Italics, bold, and italics underlined rows highlight non- (no transformed colonies), low (< 20 colonies), and best (> 1000 colonies) compatible oris, respectively

After the selection of a suitable ori, endeavors were undertaken to determine the most favorable method for exogenous DNA uptake (transformation/conjugation), further aiding in the development of *P. pantotrophus* DSM 2944 from wild-type to an rDNA host.

### Conjugation rather than electroporation is the method of choice for plasmid transfer

Several exogenous DNA uptake methods were tested to find the best approach for genetic engineering in *P. pantotrophus*. The strategies included heat shock [40], electroporation, and conjugation [34].

*P. pantotrophus* DSM 2944 cells were made chemically and electrically competent for heat shock and electroporation, respectively. The transformation process involved utilizing a fixed quantity of competent cells, which were then transformed with 100 ng plasmid DNA. Five distinct SEVA plasmids were chosen for this experiment, each carrying *P. pantotrophus-sensitive* antibiotics as the selection markers. These antibiotics included standard concentrations of the following antibiotics; ampicillin (100 mg/L), kanamycin (50 mg/L), chloramphenicol (10 mg/L), spectinomycin (100 mg/L), and tetracycline (20 mg/L). It is noteworthy that all the constructs shared common features, including a multiple cloning site and the ori RK2, with the sole variable being the antibiotic marker.

To delve deeper into potential genetic engineering techniques, we examined conjugation efficiency through direct cell-to-cell contact [41] facilitated by *pili*. The previous ori and antibiotic screening are pivotal as they determine suitable parameters for genetic engineering using conjugation. Conjugation was carried out in the presence and absence of a helper strain transformed with plasmid pRK600 carrying the F or fertility factor [42] and the plasmids pSEVA121 to 521 donor strains. Conjugation was performed using patch mating [43] (streaking all strains on top of each other). After one day, this mixed culture was plated on selective agar plates containing the respective pSEVA plasmid encoding antibiotic marker, supplemented with 50 mg/L streptomycin as a counterselection marker (only *P. pantotrophus* DSM 2944 can grow). The utilization of this dual antibiotic strategy demonstrated a straightforward and greatly efficient approach to selectively isolate recombinant *P. pantotrophus* (able to grow on both streptomycin 50 mg/l as well as pSEVA harbored antibiotic). The recombinants possessed inherent resistance to streptomycin (50 mg/L), alongside acquired resistance to the specific antibiotic present in the pSEVA plasmid used, thereby eliminating the growth of both other strains involved in the conjugation. Following 48 h, the colonies of transconjugants were quantified, serving as an assessment of the efficacy of gene transfer facilitated by conjugation into *P. pantotrophus* DSM 2944.

After 48 h of incubation at 37 °C on LB plates with respective antibiotics (Table 2), it was observed that no colonies were formed in any of the plates inoculated with chemically transformed bacteria. This suggests that a standard protocol for gram-negative bacteria is not suitable for *P. pantotrophus*. On the contrary, electroporation yielded successful transformation as *P. pantotrophus s*howed newly gained resistance on both 50 mg/L kanamycin and 100 mg/L spectinomycin. This finding also confirms that these antibiotics can be used as selection markers.

**Table 2:** Efficiency of transformation.

	Antibiotic marker	Conc. (mg/L)	Genebank seq	Transformation efficiency (cfu/µg)		Conjugation	
				Electroporation	Heat shock	Without helper strain	With helper strain
pSEVA121	Ampicillin	100	JX560322	*No colonies*	*No colonies*	*No colonies*	*No colonies*
pSEVA221	Kanamycin	50	JX560327	*2* × *10*^*4*^	*No colonies*	*No colonies*	*2* × *10*^*6*^
pSEVA321	Chloramphenicol	10	JX560332	*No colonies*	*No colonies*	*No colonies*	*1* × *10*^*1*^
pSEVA421	Spectinomycin	100	JX560337	*2* × *10*^*2*^	*No colonies*	*2* × *10*^*6*^	*2* × *10*^*8*^
pSEVA521	Tetracycline	20	JX560342	*No colonies*	*No colonies*	*No colonies*	*No colonies*

This table highlights the heat-shock and electroporation-based transformation and conjugation results of *P. pantotrophus* with five different SEVA plasmids*.* Italics underlined highlights positive and italics represnts negative transformation and conjugation results, as described by the number of colony-forming units (cfu) per microgram (µg) of added plasmid

Compared to electroporation and chemical transformation, conjugation was shown to be the best method for obtaining a maximum number of transconjugants. Although *P. pantotrophus* DSM 2944 was found to be capable of performing conjugation without the helper plasmid, the number of recombinant clones increased 100-fold with the aid of the helper plasmid pRK600 in the case of plasmid pSEVA421.

Since transforming *P. pantotrophus* DSM 2944 was found to be most effective with conjugation [1], which is a key step in the construction of a novel SynBio chassis, all subsequent DNA modification experiments were conducted using this method.

Post establishment of successful gene transfer techniques, the following investigations were conducted to depict the strain's fitness or stability as a suitable host for maintaining a specific plasmid under non-selective conditions. This marks the completion of *P. pantotrophus* as a rDNA host and marks the beginning of becoming a successful SynBio chassis.

### Novel chassis *P. pantotrophus* DSM 2944 is capable of plasmid maintenance

Plasmid stability in *P. pantotrophus* DSM 2944 under non-selective conditions was tested [44]. Low copy number RK2-based vector pSEVA221 was selected to determine host fitness. As a control, *E. coli* DH5α containing pSEVA221 was used [26]. Both strains were grown in antibiotic-free LB medium in shake flasks and passaged on to the next shake flask using the same conditions daily. An aliquot was plated on LB-agar containing the selective marker (kanamycin 50 mg/L) during each passaging. For every sub-culturing, the number of transferred cells was kept constant, and sub-culturing was performed after the cells ceased to divide.

**Fig. 4:**
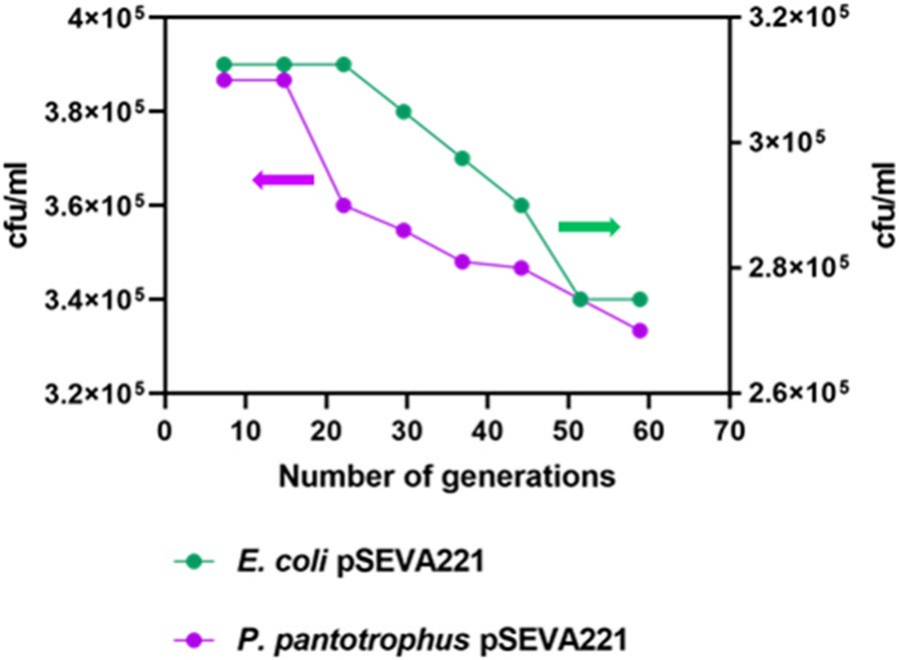
Test of plasmid maintenance. *P. pantotrophus* DSM 2944 and *E. coli* DH5α were tested for stable plasmid maintenance without selection pressure

The comparative analysis between the established chassis *E. coli* and the emerging chassis *P. pantotrophus* (Fig. 4) demonstrates similar plasmid maintenance using pSEVA221 in the absence of antibiotic selection (kanamycin 50 mg/L). In the case of *E. coli*, the cfu/mL decreases at a steady rate from generation 22 (3.9*10^5^ cells) to 44 (3.6*10^5^ cells). On the other hand, *P. pantotrophus* experiences a more rapid reduction, with a substantial decline of 200 cfu/mL occurring between generations 14 and 22. Nonetheless, when considering the overall decrease of host cells carrying the pSEVA221 plasmid throughout 60 generations, the difference in cfu/mL between *E. coli* (3.9*10^5^ to 3.4*10^5^) and *P. pantotrophus* (3.1*10^5^ to 2.7*10^5^) is merely 100 cfu/mL. This inference shows that *P. pantotrophus* DSM 2944 can maintain plasmids [45, 46], and thus can be employed for varied plasmid-based applications. After ensuring the establishment of plasmid stability, an examination of synthetic promoters on gene expression was conducted.

### Synthetic promoters for tailored gene expression

**Fig. 5:**
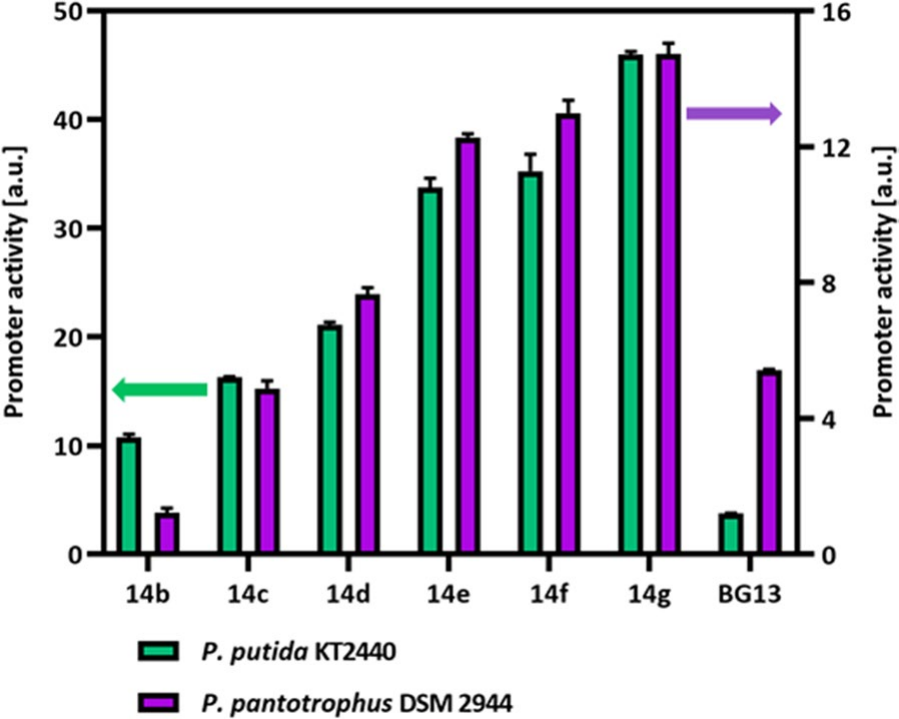
Characterization of seven synthetic promoters in *P. pantotrophus* DSM 2944 in comparison to *P. putida* KT2440. Triplicates of every strain were cultured in the BioLector (Bechmann Coulter GmbH, Aachen, Germany) in MSM medium with 20 mM glucose in a 96-well plate. Promoter activity was calculated from the slope of GFP fluorescence to optical density during the exponential phase. Error bars indicate the deviation from the mean of the three replicates

The mini-Tn7-based promoter transposon system originally constructed for *P. putida* KT2440, consisting of the synthetic constitutive promoters BG14x (x = b, c, d, e, f, and g) and BG13 [47, 48], was used. Although the Tn7-based system possesses an advantage over plasmid-based systems, the former can prove inadequate due to the significant variation in translation efficiency contingent upon the non-coding 5′ region sequence of the gene of interest (GOI) [49, 50]. This challenge can be addressed by implementing translational couplers in a bicistronic design as described in [51, 52]. The constructs containing a gene coding for superfolder green fluorescent protein (*msf*GFP) [53] under the control of the different promoters with kanamycin as the selection marker were maintained as vectors in *E. coli* PIR2 (Table 3) with RK6 origin that cannot replicate in *P. pantotrophus.* Hence the target promoter-containing construct is chromosomally integrated into the *att*Tn7 site (Fig. 5).

**Table 3:** Plasmids and strains used in this study

Strains and plasmids	Description	References
**Plasmids**		
pSEVA211	KmR, ori R6K, standard multiple cloning site	Lorenzo et al. [26]
pSEVA221	KmR, ori RK2, standard multiple cloning site	Lorenzo et al. [26]
pSEVA231	KmR, ori pBBR1, standard multiple cloning site	Lorenzo et al. [26]
pSEVA241	KmR, ori pRO1600/ColE1, standard multiple cloning site	Lorenzo et al. [26]
pSEVA251	KmR, ori RFS1010, standard multiple cloning site	Lorenzo et al. [26]
pSEVA261	KmR, ori p15A, standard multiple cloning site	http://seva.cnb.csic.es
pSEVA271	KmR, ori pSC101, standard multiple cloning site	http://seva.cnb.csic.es
pSEVA281	KmR, ori pUC, standard multiple cloning site	http://seva.cnb.csic.es
pSEVA291	KmR, ori pBR322/ROP, standard multiple cloning site	http://seva.cnb.csic.es
pSEVA121	Apr, ori RK2, standard multiple cloning site	Lorenzo et al. [26]
pSEVA321	CmR, ori RK2, standard multiple cloning site	Lorenzo et al. [26]
pSEVA421	Sm/SpR, ori RK2, standard multiple cloning site	Lorenzo et al. [26]
pSEVA521	TcR, ori RK2, standard multiple cloning site	Lorenzo et al. [26]
pRK600	CmR, ori ColE1, tra + mob + of RK2	Boyer et al. [77]
pEMG	KmR, oriR6K, lacZα with two flanking I-SceI sites	Lorenzo et al. [56]
pSW2	GmR, oriRK2, xylS, Pm → I sceI (transcriptional fusion of I-sceI to Pm)	Yang et al. [78]
pBG14b FRT Kan	KmR, GmR, oriR6K, pBG-derived, promoter 14b, msfGFP	Zobel et al. [51]
pBG14b FRT Kan	KmR, GmR, oriR6K, pBG-derived, promoter 14c, msfGFP	Zobel et al. [51]
pBG14c FRT Kan	KmR, GmR, oriR6K, pBG-derived, promoter 14d, msfGFP	Zobel et al. [51]
pBG14d FRT Kan	KmR, GmR, oriR6K, pBG-derived, promoter 14d, msfGFP	Zobel et al. [51]
pBG14e FRT Kan	KmR, GmR, oriR6K, pBG-derived, promoter 14e, msfGFP	Zobel et al. [51]
pBG14f FRT Kan	KmR, GmR, oriR6K, pBG-derived, promoter 14g, msfGFP	Zobel et al. [51]
pBG14g FRT Kan	KmR, GmR, oriR6K, pBG-derived, promoter 14g, msfGFP	Zobel et al. [51]
pBG13 FRT Kan	KmR, GmR, oriR6K, pBG-derived, promoter Pem7, msfGFP	Zobel et al. [51]
pBG_*pcaRtphA2A3BA1pcaK*	KmR, oriR6K, pBG-derived, *tph* operon	Nugraha et al. [58]
p∆PHB	KmR, oriR6K, carrying TS1 and TS2 elements each 1000 bp upstream and downstream respectively of PHB cassette in *P. pantotrophus* DSM 2944	This study
**Strains**		
***Escherichia coli***		
DH5α	supE44, 1lacU169 (Φ80lacZ1M15), hsdR17 (rK − mK +), recA1, endA1, thi-1, gyrA96, relA1	Hanahan et al. [79]
Top10	mcrA, Δ(mrr-hsdRMS-mcrBC), Phi80lacZ(del)M15, ΔlacX74, deoR, recA1, araD139, Δ(ara-leu)7697, galU, galK, rpsL(SmR), endA1, nupG	Invitrogen
CC118	F- LAM- araD139 DE(ara leu)7697 DE(lacX74) phoA20(del) galE galK thi rpsE rpoB argE(Am) recA1	Invitrogen
DH5*α*λpir	endA1 hsdR17 glnV44 (supE44) thi-1 recA1 gyrA96 relA1 φ80dlacΔ(lacZ)M15 Δ(lacZYA-argF)U169 zdg-232::Tn10 uidA::pir +	Platt et al. [80]
HB101	F − mcrB mrr hsdS20(rB − mB −) recA13 leuB6 ara-14 proA2 lacY1 galK2 xyl-5 mtl-1 rpsL20(SmR) gln V44 λ −	Hanahan et al. [79]
pTnS-1	ApR, ori R6K, TnSABC + D operon	Choi et al. [81]
PIR2	F- Δlac169 rpoS (Am) robA1 creC510 hsdR514 endA reacA1 uidA (ΔMLui)::pir	Choi et al. [81]
pSW1	psw-1: ApR, oriRK2, xylS, Pm → I-sceI (transcriptional fusion of I-sceI to Pm)	Life Technologies
***Pseudomonas putida* KT2440**		
WT	Wild type	Nelson et al. [82]
BG14b FRT Kan	KmR with FRT flanking sequences, *P. putida* KT2440 with genomic insertion of pBG14b FRT Kan	Köbbing et al. [48]
BG14c FRT Kan	KmR with FRT flanking sequences, *P. putida* KT2440 with genomic insertion of pBG14c FRT Kan	Köbbing et al. [48]
BG14d FRT Kan	KmR with FRT flanking sequences, *P. putida* KT2440 with genomic insertion of pBG14d FRT Kan	Köbbing et al. [48]
BG14e FRT Kan	KmR with FRT flanking sequences, *P. putida* KT2440 with genomic insertion of pBG14e FRT Kan	Köbbing et al. [48]
BG14f FRT Kan	KmR with FRT flanking sequences, *P. putida* KT2440 with genomic insertion of pBG14f FRT Kan	Köbbing et al. [48]
BG14g FRT Kan	KmR with FRT flanking sequences, *P. putida* KT2440 with genomic insertion of pBG14g FRT Kan	Köbbing et al. [48]
BG13 FRT Kan	KmR with FRT flanking sequences, *P. putida* KT2440 with genomic insertion of pBG13 FRT Kan	Köbbing et al. [48]
***Paracoccus pantotrophus* DSM 2944**		
WT	Wild type	Bockwoldt et al. [24]
∆PHB	*P. pantotrophus* strain with the entire polyhydroxyalkanoate cassette deleted, unable to produce PHB	This study
*tph*	*P. pantotrophus* strain with the *tph* operon integrated at the attn7 site	This study
TA static *tph*	*P. pantotrophus* strain with the *tph* operon evolved in terephthalic acid	This study
dynamic *tph*	*P. pantotrophus* strain with the *tph* operon evolved in alternating carbon sources of ethylene glycol and terephthalic acid	This study
*tph* EG static	*P. pantotrophus* strain with the *tph* operon evolved in ethylene glycol	This study

The resulting *P. pantotrophus* DSM 2944 strains, each regulated by distinct promoters controlling *msf*GFP expression, exhibited a consistent pattern in terms of promoter strength. Comparative analysis with the *P. putida* KT2440 control, specifically in the context of the BG14x system, revealed a hierarchy of expression levels among the promoters. Notably, the pBG14b promoter demonstrated the weakest expression, while the pBG14g promoter displayed the strongest, resulting in an overall approximately three-fold reduction in strength (46 vs. 15, respectively). Interestingly, promoter BG13, which exhibited the lowest activity in *P. putida* KT2440, had a nearly twofold increase in promoter activity when it was introduced into *P. pantotrophus* DSM 2944. The above promoter expression profile demonstrates the application of cross-species regulatory elements (promoters) as tools for genetic engineering. Thereby showcasing the applicability of transcription tuning with the help of synthetic promoters originally designed for *P. putida* KT2440 and bicistronic design as a promising strategy to overcome expression rate challenges, adjust enzyme concentrations, modulate metabolic fluxes [54], and improve production of recombinant proteins [55]. Thus, through the incorporation of regulatory elements within *P. pantotrophus* DSM 2944, the strain’s transition from an rDNA host to a SynBio chassis was achieved.

### Gene deletion protocol that facilitates the elimination of PHB production

The technique of precise gene deletion using the pEMG platform, developed by Martinez-et al. for *P. putida* KT2440 was adapted to *P. pantotrophus* DSM 2994 [56]. Here, the PHB operon was selected as proof-of-concept gene deletion. The pEMG vector was constructed with the I-SceI sites flanking 1,000 bp each upstream and downstream of the PHB cassette consisting of the four genes *phaR*, *phaP*, *phaC*, and *phaZ* [24].

**Fig. 6:**
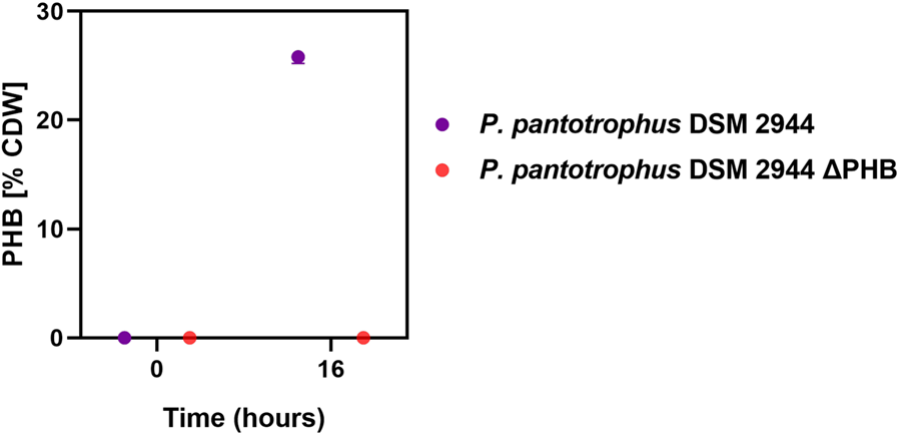
Polyhydroxybutyrate (PHB) cassette deletion. Both *P. pantotrophus* DSM 2944 WT and the gene deletion strain ∆PHB were grown in MSM medium supplemented with 40 mM of glucose and nitrogen limitation. Cultivation conditions included growth in shake flasks at 37 °C with 200 rpm. Harvested and extracted biomass was used for PHB content [% CDW] quantification after 16 h. The experiment was done in triplicates and error bars indicate deviation from the mean

Strain *P. pantotrophus* DSM 2944 ∆PHB produced no PHB, while the WT consisted of 25% PHB (Fig. 6), thus confirming successful gene deletion (with a success rate of 98%). This concluded the successful application of the pEMG-based deletion technique in *P. pantotrophus* DSM 2944 and asked for the introduction of heterologous genes into the genome.

### Gene integration enables the metabolization of PET monomers

To advance the genetic toolbox of the SynBio chassis, a conscious effort was made to align these endeavors with sustainability objectives. *P. pantotrophus* DSM 2944, known for its broad substrate utilization, naturally possesses the ability to utilize the PET monomer ethylene glycol as the sole carbon source [19]. Here, terephthalic acid (TA) catabolism was engineered to complement the native ability.

**Fig. 7:**
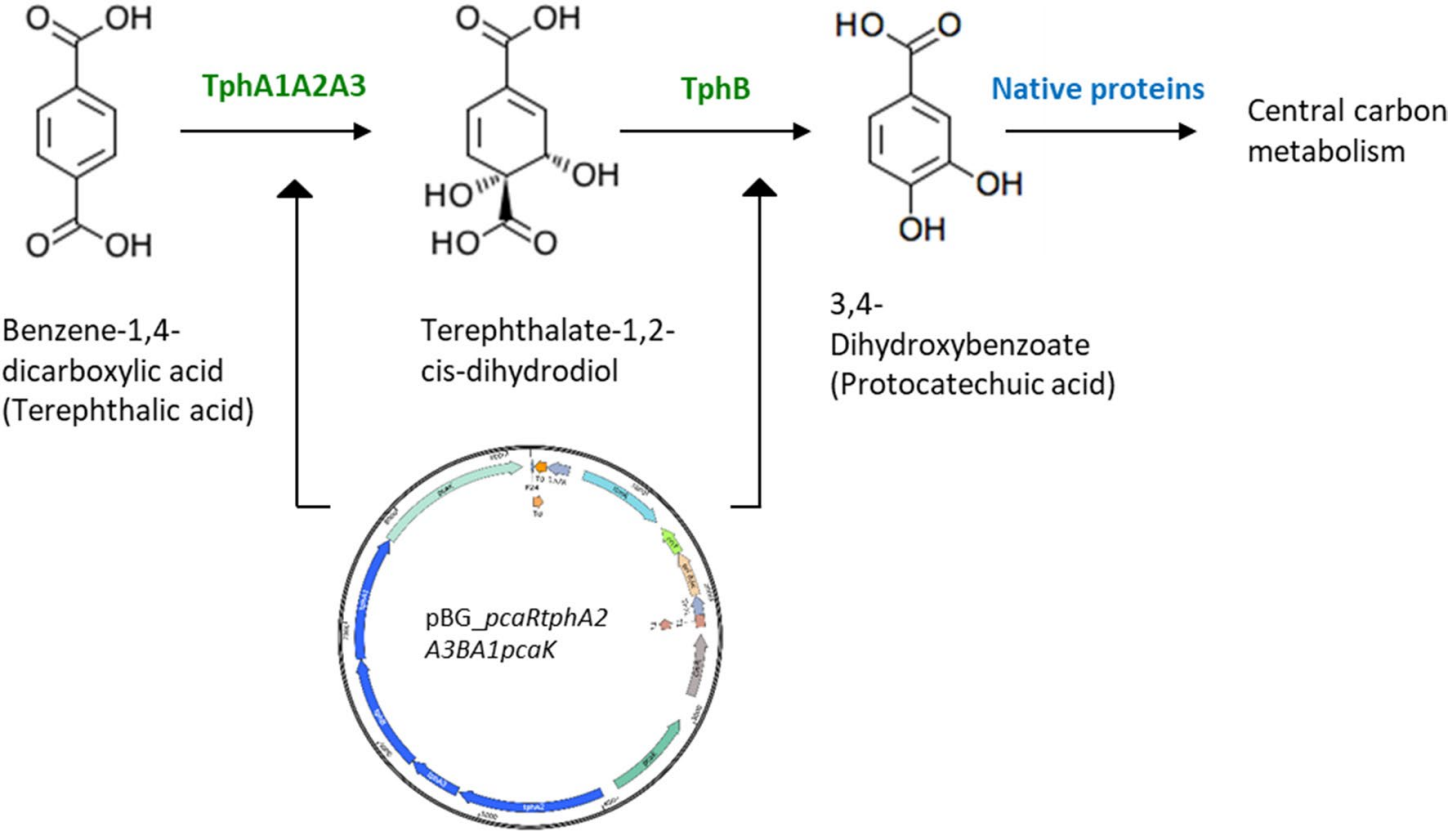
Schematic representation of terephthalic acid metabolism. The proteins marked in green (TphA1A2A3 and TphB) are heterologous and encoded on the plasmid while the strain’s native pathway is marked in blue. Moreover, heterologous proteins, PcaR encoding regulator function, and PcaK encoding cytoplasmic TA transporter are not marked in the above picture

Fortunately, the strain could already grow on protocatechuic acid (PCA) or 3,4-dihydroxybenzoic acid, an intermediate molecule for terephthalic acid degradation (Fig. 7) [57]. Henceforth, the introduction of the *tph* cassette (rDNA) comprising genes encoding the proteins PcaR, TphA2, A3, A1, B, and PcaK (encoding transporter for TA) was performed using the Tn7-based plasmid pBG_*pcaRtphA2A3BA1pcaK* [58].

**Fig. 8:**
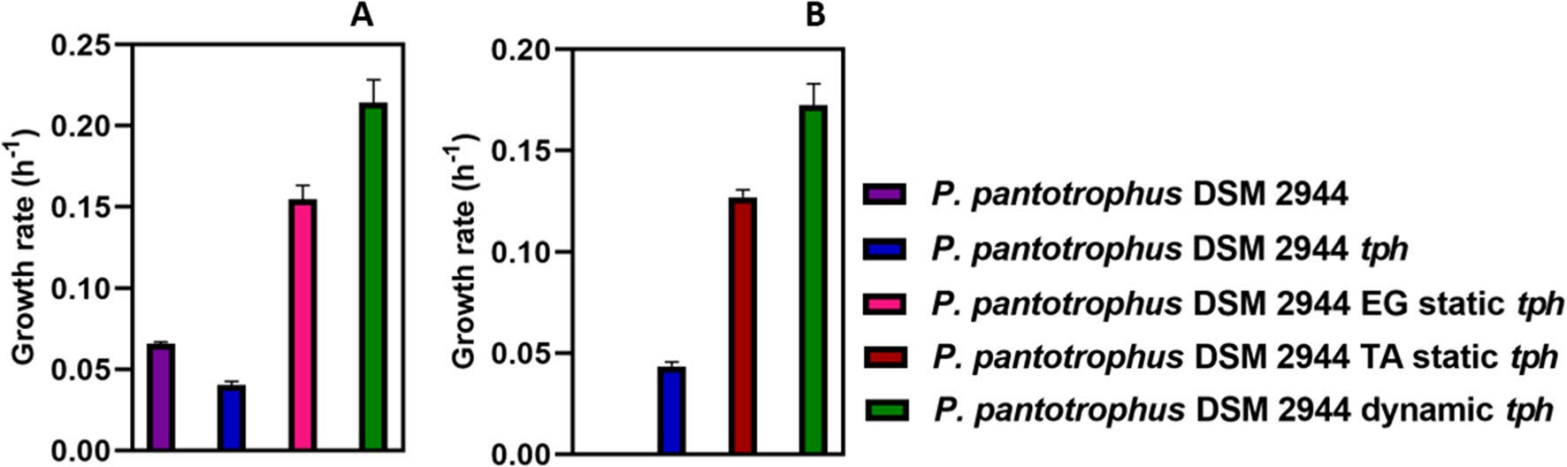
Adaptive laboratory evolution results on PET monomers. Comparison of the wild type versus non-evolved and evolved strains of *P. pantotrophus* DSM 2944 *tph* on A being 40 mM ethylene glycol (EG) and B being 20 mM of terephthalic acid (TA) as the sole carbon source. The Growth Profiler was used for online biomass measurements. The experiment was conducted in triplicates, and the error bars represent the deviation from the mean

The resulting recombinant *P. pantotrophus* DSM 2944 *tph* was able to grow on both monomers of PET (EG and TA) (Fig. 8), thus displaying the metabolic versatility of *P. pantotrophus* DSM 2944 and its applicability as a chassis microorganism for biotechnology. These experiments conclude the successful genome engineering (deletion and integration) as part of the genetic toolbox and represent establishing *P. pantotrophus* DSM 2944 as a novel chassis organism. To further streamline and improve the substate uptake of PET monomers, the newly developed SynBio chassis was subjected to adaptive laboratory evolution, thereby showcasing ALE as another tool for metabolic engineering (Fig. 8).

### Adaptive laboratory evolution to enhance growth and utilization of PET monomers

ALE was performed with the recombinant strain *P. pantotrophus* DSM 2944 *tph*. Two types of ALE strategies were carried out, comprising static (adaptation using one substrate) and dynamic (adaptation using alternating substrates) ALE [59]. Ultimately, the strain with the highest growth rate was tested for substrate consumption (Fig. 8).

**Fig. 9:**
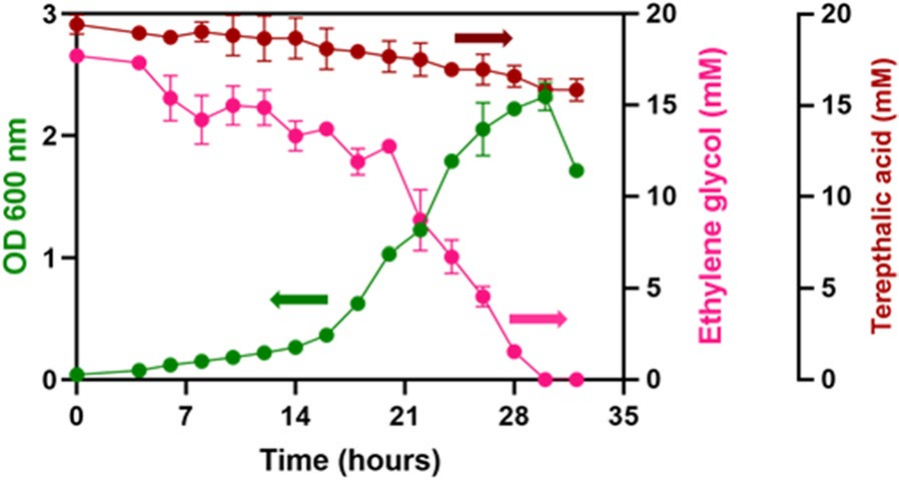
PET monomer utilization. The line graph depicts the growth (OD_600_) of *P. pantotrophus* DSM 2944 dynamic *tph* on both ethylene glycol (EG) and terephthalic acid (TA). The cultivation was performed for 34 h in MSM medium supplemented with equimolar concentrations of EG and TA (20 mM) in polypropylene square 24-deep well microplates at 37 °C with 2 mL of medium (20% v/v) for optimal oxygen transfer rate [60]

*P. pantotrophus* DSM 2944 showed enhanced growth on the PET monomers after 120 generations of ALE. The isolated mutants grow faster than the wild-type or non-evolved counterparts. The growth rate observed when using EG as the exclusive carbon source reveals intriguing outcomes, particularly showcasing an ascending trend in growth rates: starting from the wild-type *P. pantotrophus* DSM 2944 (0.07 h^−1^), advancing to the evolved variant *P. pantotrophus* DSM 2944 EG static *tph* (cultured solely in EG) (0.15 h^−1^), and culminating in the highest growth rate with *P. pantotrophus* DSM 2944 dynamic *tph* (evolved under alternating EG and TA conditions) (0.21 h^−1^). The incorporation of the heterologous *tph* cassette appeared to negatively influence the growth rate on EG, as indicated by the slower growth rate of *P. pantotrophus* DSM 2944 tph (0.04 h^−1^) when contrasted with the wild-type strain. In the context of utilizing TA as the sole carbon source, a comparable pattern in growth rate emerges. The non-adapted *P. pantotrophus* DSM 2944 *tph* strain exhibits the lowest growth rate (0.04 h^−1^), succeeded by TA static *tph* (cultivated exclusively in TA) (0.13 h^−1^), while the dynamic *tph* strain demonstrates the highest growth rate (0.17 h^−1^). Despite 20 mM TA having four times the carbon per mole compared to EG, the observed lowered growth rates in TA compared to EG strongly indicate that the strains did not fully utilize the entire substrate during growth. These results motivated us to perform a more comprehensive investigation to determine the assimilation of PET monomers by the adapted *P. pantotrophus* DSM 2944 dynamic *tph*, featuring enhanced growth rates on both monomers (Fig. 8). The strain was cultivated utilizing equimolar concentrations (20 mM) of EG and TA, as carbon sources (Fig. 9).

It was evident that EG was fully consumed within 30 h. In contrast, the concentration of TA decreased by approximately 4 mM, with the remaining portion of the substrate persisting in the medium. This can arise from the fact that the engineered strain prefers to consume EG over TA, being the substrate, it can degrade naturally.

In conclusion, the outcomes highlight the application of the genetically modified *P. pantotrophus* DSM 2944 dynamic *tph* strain in utilizing both PET monomers, as carbon sources and highlighting a successful application of the genetic toolbox for the development of the novel SynBio chassis.

## Discussion

This study is centered on transforming *P. pantotrophus* DSM 2944, an environmental isolate from waste effluent treatment, into a metabolically and physiologically resilient SynBio chassis. The following discussion highlights existing studies that revolve around the genus *Paracoccus* and is used to compare the findings from this study to provide a comprehensive overview.

As previously mentioned, genetic robustness in a chassis necessitates freedom from reliance solely on strain-specific plasmids. An instance of such strain specificity is evident in plasmid pWKS1 for *P. pantotrophus* DSM 11073 [61]. Characterized as a cryptic plasmid, conveying no overt phenotype to the host cell and harboring genes with unidentified attributes, it constrains the applicability of cross-species gene transfer techniques. Whereas the novel chassis, belonging to the above species, possesses the capability to use plasmids having different oris, thus removing the dependency of strain-specific plasmids and making genetic manipulations on *P. pantotrophus* DSM 2944 simpler.

Taking an alternative viewpoint, the utilization of frequently employed plasmids like pUC19, known for its capacity to generate a high copy number, demonstrated effective expression of cloned genes in *Paracoccus sp.* SY [62]. But on the contrary, the novel chassis *P. pantotrophus* DSM 2944 was unable to replicate pUC19. It was found that low copy number ori RK2 (broad-range plasmid affiliated with the incP incompatibility group) [63] to be the most compatible ori. This is in agreement with previous results from *P. denitrificans* [64], thus showcasing the similarity and dissimilarities in plasmid preferences between members of the genus *Paracoccus*. Preference for low copy number oris can be explained by the metabolic burden where the cell prioritizes high amounts of protein production over essential metabolic reactions crucial to the host cell [65, 69].

In the light of efficient gene transfer techniques, where it was shown that strain *P. denitrificans* is capable of undergoing electroporation [66], this study provides further information through testing of several high- and low-copy number plasmids and their related transformation efficiencies. Interestingly, although no *tra* genes (coding for conjugational transfer) were reported in *P. pantotrophus* DSM 2944, colony formation was observed when spectinomycin at a concentration of 100 mg/L, was used as a selection marker for the selection of transconjugants. This phenomenon can be explained with the help of a previous study that portrays gene *aadA* (GeneID: 1,252,782) [67] encoding protein aminoglycoside (3'') (9) adenylyltransferase. This enzyme was found to be responsible for providing resistance to aminoglycoside antibiotics including streptomycin, and spectinomycin was found in *P. pantotrophus* DSM 2944, with an 80% (BLAST) nucleotide similarity. This can justify the gained resistance to spectinomycin when the strains were subjected to the antibiotic, thereby explaining the observation of positive *P. pantotrophus* clones in the selection plate containing 100 mg/L spectinomycin. A previous study highlighted that both *Rhodobacter sphaeroides* and *P. denitrificans*, belonging to the same family (Rhodobacteraceae), were capable of replicating the same plasmids [68]. Based on these findings, this study expands the concept further by showcasing the application of several different plasmids (both SEVA and Tn7-based) in different classes of microorganisms: The Alphaproteobacterium *P. pantotrophus* DSM 2944 and Gammaproteobacteria (*E. coli*, *P. putida*). This showcases the broad applicability of the selected plasmids and the genetic robustness of the chosen chassis.

The gene deletion system pEMG was originally developed for *P. putida* KT2440 and successfully applied in related species, such as *Pseudomonas protegens* [69], *Pseudomonas aeruginosa* [70], *P. umsogensis*, *P. taiwanensis*, and others. It was here used successfully to delete the genes coding for PHB-producing enzymes. This pivotal accomplishment holds notable importance, marking the first application of the pEMG system within the *Paracoccus* genus and opening doors for further genome reduction in *Paracoccus*.

Existing studies involving the application of a Tn5-based transposon system [71], as a genetic tool for gene insertion were already established in *P. denitrificans* PD1222 [72]. One of the key factors using transposon-based molecular applications is the uniform distribution of transposon insertion [73]. Tn5 transposon is found to randomly integrate into the target genome [74]. To expand the metabolic versatility of *P. pantotrophus* DSM 2944 the introduction of the terephthalic acid catabolic pathway by the encoding operon was performed using the Tn7 system. Although the insertion of this operon resulted in the slight consumption of TA, *P. pantotrophus* DSM 2944 *tph* was further expedited for growth on TA using adaptive laboratory evolution. Thus, to make the novel chassis *P. pantotrophus* DSM 2944 reach high growth rates such as *P. putida* TDM461 (0.72 h^−1^ on 10 mM TA as carbon source) [75], further studies need to be performed. One hint could be the potential issue with TA permeability across the cell membrane. To address this limitation and enhance TA consumption, the solution could be the incorporation of the *tphC* gene. This gene encodes the solute-binding protein TphC, which is responsible for binding TA and facilitating its transport via the transmembrane transport proteins TpiA and TpiC [76].

## Conclusion

Here, *P. pantotrophus* DSM 2944 was promoted from a wild-type strain with interesting metabolic properties to a SynBio chassis by following the chassis construction guidelines laid out by Calero and Nickel [7] and de Lorenzo [1].

Key findings included the identification of broad-range ori RK2 as a convenient tool for plasmid-based gene expression. Notably, the strain exhibited promising plasmid stability without antibiotics. Transformation methods were explored, highlighting the efficiency of electric pulse-based transformation and the ineffectiveness of calcium chloride-based methods. Conjugation emerged as the most effective method, further improved by a helper plasmid (pRK600). The toolbox extended to targeted scar-less gene deletion (*P. pantotrophus* DSM 2944 ΔPHB) [56] and Tn7-based GFP-tagged promoter systems for gene expression [51]. Native resistance was investigated employing a dual antibiotic-based combination, comprising kanamycin and streptomycin, to facilitate uncomplicated selection during conjugational gene transfer. Beyond the double-antibiotic selection approach, alternative selection parameters can be explored by leveraging the extensive metabolic repertoire of *P. pantotrophus* DSM 2944. This repertoire encompasses diverse carbon sources such as formate, acetate, and ethylene glycol, along with nitrogen sources encompassing various amino acids. Notably, l-histidine emerges as a prominent nitrogen source, exhibiting a growth rate of 0.73 h^−1^.

Furthermore, the introduction of the TA operon led to the creation of strain *P. pantotrophus* DSM 2944 *tph* and this strain demonstrated growth on both EG (complete consumption) and TA (low consumption) as carbon sources, highlighting PET consumption. Lastly aided with an adaptive evolution strategy, enhanced growth rates on both PET monomers were shown to be possible, signifying the capability of this new chassis strain toward genetic modifications and improved functionality. The culmination of this work not only expands *P. pantotrophus* DSM 2944 as a new SynBio but beckons further research to propel it into a Standardized SynBio chassis [1], in the future. The contributions shown here may motivate researchers to choose the metabolically interesting genus *Paracoccus* for their endeavors.

## Materials and methods

### Bacterial strains and growth media

The chemicals utilized in this study were acquired from Carl Roth (Karlsruhe, Germany), Sigma-Aldrich (St. Louis, MO, USA), or Merck (Darmstadt, Germany) unless specified otherwise. The wild-type *Paracoccus pantotrophus* DSM 2944 [24] was procured from the German Collection of Microorganisms and Cell Cultures (Braunschweig, Germany). Except for experiments utilizing specific carbon sources, both the wild-type *P. pantotrophus* and genetically modified strains were cultivated in Lysogeny broth (LB) consisting of 10 g tryptone, 5 g yeast extract, and 5 g of NaCl per liter of deionized water, serving as a complex medium, incubated at 37°C. Agar at a concentration of 2% was added to media requiring solidification. Furthermore, both the wild-type as well as genetically modified strains of *P. pantotrophus* were also cultivated in delft mineral salt medium (MSM) containing 3.88 g of K_2_HPO_4_, 1.63 g of NaH_2_PO_4_, 2.00 g of (NH_4_)_2_SO_4_, 0.1 g of MgCl_2_ × 6 H_2_O, 10 mg of EDTA, 2 mg of ZnSO_4_ × 7 H_2_O, 1 mg of CaCl_2_ × 2 H_2_O, 5 mg of FeSO_4_ × 7 H_2_O, 0.2 mg of Na_2_MoO_4_ × 2 H_2_O, 0.2 mg of CuSO_4_ × 5 H_2_O, 0.4 mg of CoCl_2_ × 6H_2_O, and 1 mg of MnCl_2_ × 2 H_2_O per liter of water to evaluate growth on a sole carbon source.

### Bacterial growth quantification and rate determination

The optical density was measured using an Ultrospec 10-cell density meter (Amersham Biosciences, UK) at _600_ nm. An OD_600_ of 1.0 corresponded with a cell dry weight of 366 mg L^−1^.

Furthermore, to enable real-time and automated monitoring of bacterial growth and growth rate [85], the Growth Profiler 960 (System Duetz, EnzyScreen BV, Heemstede, The Netherlands) was employed. Throughout the cultivations in the Growth Profiler, a constant temperature of 30°C and a shaking speed set to 250 rpm (revolutions per minute) were maintained. The cultivation was carried out in 96-well MTP plates (CR1496dg: polystyrene white square 96-half-deep well microtiter plates), which were covered with sandwich covers (CR1396: universal sandwich cover for 96-well MTPs). The entire run spanned 48 h.

The Growth Profiler recorded online green values, which were subsequently converted to OD_600_ values, depicting the growth of the bacteria [86]. To calculate growth rates, a MATLAB-based script was utilized in the analysis of the recorded data, which portrays exponential growth [87].

### Cloning and strain engineering

The plasmids were constructed using Gibson assembly [88] with the NEBuilder HiFi DNA Assembly Master Mix (New England Biolabs GmBH, Frankfurt, Germany). All the designed primers used in this study were manufactured as unmodified DNA oligonucleotides from Eurofins Genomics (Ebersberg, Germany). Q5 High-Fidelity DNA Polymerase was used for all Polymerase Chain Reaction (PCR) products over 2000 bp, while smaller fragments were amplified using One-Taq DNA Polymerase (New England Biolabs GmBH, Frankfurt, Germany). Whereas in fragments with high GC content, Q5 High GC Enhancer (New England Biolabs GmBH, Frankfurt, Germany) was supplemented with the PCR reaction mix. In-depth information about used strains and plasmids can be found in Table 3 and about oligonucleotides in Table 4.

**Table 4:** Oligonucleotides used in this study

Primer	Sequence (5’ → 3’)	Function	Refs.
UP01	ccgcgctggaggatcatccaTGAGAAGCCTCGCCTTCC	Amplification TS1 fragment, p∆PHB	This work
UP02	gccgggtttcGAGAACTGCGTCTTGCGC	Amplification TS1 fragment, p∆PHB	This work
UP03	cgcagttctcGAAACCCGGCGCGGCCAA	Amplification TS2 fragment, p∆PHB	This work
UP04	tcgttttccgggacgccggcGCCGGCGGGATAGTCGCG	Amplification TS2 fragment, p∆PHB	This work
UP05	GCCGGCGTCCCGGAAAAC	Amplification pEMG vector	This work
UP06	TGGATGATCCTCCAGCGCG	Amplification pEMG vector	This work
UP07	GTGCTGCAAGGCGATTAAGT	Control p∆PHB construct	This work
UP08	GCTAAAGCTGGAACGGGGAA	Control p∆PHB construct	This work
UP09	GCCCTTCCATGAATTGCGTC	Control p∆PHB construct	This work
UP10	GACCACCAAGCGAAACATCG	Control p∆PHB construct	This work
UP11	CGGTAGCCGCTTACTTGGC	Control of triparental mating knockout	This work
UP12	GCTATGACCATGATTACGCCGG	Control of triparental mating knockout	This work
UP13	GACGTTGTAAAACGACGGCC	Control of triparental mating knockout	This work
UP13	ACCTCGGCCGCAGTGAT	Control of triparental mating knockout	This work
UP14	TCTTCTTGCTAGGCGGGTTG	Verify *P. pantotrophus* DSM 2944 ∆PHB knockout	This work
UP15	GACGATTACGTCAGCGCCTA	Verify *P. pantotrophus* DSM 2944 ∆PHB knockout	This work
8F	AGAGTTTGATCCTGGCTCAG	16S Fwd	[83]
1492R	CGGTTACCTTGTTACGACTT	16S Rev	[84]

The newly assembled vector constructs were transformed into chemically competent *E. coli* PIR2 cells using a heat shock protocol [79]. Gene transfer in *P. pantotrophus* was conducted using either transformation using electroporation or conjugation as described by Wynands et al. [34]. The efficiency of transformation (cfu/µg) was calculated as described in (https://www.edvotek.com/how-to-calculate-transformation-efficiency). To assess *P. pantotrophus* DSM 2944's capability for direct cell-to-cell gene transfer via conjugation, mating experiments were conducted under two conditions: one with a helper strain carrying plasmid pRK600, facilitating the conjugational transfer, and another without. Donor strains carried plasmids (pSEVA121, 221, 231, 241, 251) with specific antibiotics as selection markers. Transconjugants were identified by positive growth on antibiotic plates with the requisite markers.

The pEMG genomic deletion was executed through homologous recombination involving plasmid p∆PHB and *P. pantotrophus* DSM 2944, facilitated by plasmid pSW2 expressing the I-SceI enzyme via conjugation. The selection of desired recombinant clones was accomplished utilizing the appropriate TS1 and TS2 primers (refer to Table 3). The resulting deleted strain, denoted as *P. pantotrophus* DSM 2944 ∆PHB, exhibited the absence of polyhydroxybutyrate (PHB) production, as achieved through the pEMG deletion method detailed in [56]. In contrast, heterologous gene integration into the genome was achieved using a Tn7 transposon-based system, as illustrated in [81], positioning the requisite genes downstream of the *glmS* gene [89] in *P. pantotrophus* DSM 2944. Conjugation was performed by the *E. coli* donor strain holding the respective pBG-plasmid, the helper strain *E. coli* HB101 pRK600, *E. coli* DH5α λpir pTnS1 providing the required transposase, and the recipient, *P. pantotrophus* DSM 2944.

The amplified PCR products were gel-extracted with a DNA Gel Extraction kit (New England Biolabs, Ipswich, Massachusetts, USA). The concentration of purified fragments was measured with a NanoDrop One (Thermo Scientific, Waltham, Massachusetts, USA). Colony PCR was performed to either amplify DNA fragments from the genome or verify the recombinant strain using 16S sequencing. Finally, all required fragments were sequenced using the Mix2Seq service from Eurofins Genomics (Ebersberg, Germany).

### Fluorescent measurement and determination of promoter activity

The activity of the synthetic promoters was determined by the intensity of the msfGFP [90]. Fluorescence was quantified using the Biolector (M2P Labs, Baesweiler, Germany) where the excitation and the emission wavelength were set to 488 and 520 nm respectively, along with a gain of 40. Tested strains included *P. putida* KT2440 [47, 48] as the control and newly constructed *P. pantotrophus* DSM 2944 both having expression cassettes integrated into the genome. The cells were grown at 30°C with a shaking speed of 200 rpm on MSM media supplemented with 20 mM glucose as the sole carbon source. To omit the influence of varying cell numbers on fluorescence measurement, the starting OD_600_ of all the cells for the experiment was set to 0.01. A calibration between OD_600_ and fluorescence was conducted for both strains. Finally, the promoter activity is determined by calculating the slope of GFP fluorescence to optical density during the exponential phase [47].

### Adaptive laboratory evolution

Adaptive laboratory evolution was performed on *P. pantotrophus* DSM 2944 *tph*, to obtain phenotypes with improved substrate-utilizing capabilities. Two adaptive laboratory evolution techniques were employed: static ALE involved continuous sub-culturing with a fixed carbon source (EG or TA), while in dynamic ALE alternated substrates [59] were used. Delft minimal medium supplemented with 40 mM EG or 20 mM TA was used for all experiments. The strains were grown in polypropylene square 24-deep well microplates at 37°C. OD_600_ was measured every 24 h for cells grown in EG and 48 h in terephthalic acid and the cells were sequentially transferred to a fresh medium with a starting OD_600_ of 0.03. Sub-culturing was carried out for 21 days. After this period, the adapted strains were streaked out on 20 mM TA plates to obtain single isolates, which were subsequently tested for improved growth in the Growth Profiler.

### Polyhydroxyalkanoate quantification

Pre-weighed 10 mg of lyophilized cell biomass of the required strain were mixed with a (1:1) ratio of acidified methanol and chloroform in heat-resistant Pyrex tubes. To this mixture, 10 μl of tridecanoic acid (in 20 g L^−1^ in ethanol) was added as the internal standard. The mixture was vortexed, incubated at 100 °C for 2 h, and then cooled. After cooling, autoclaved distilled water was added and centrifuged, and finally, the organic phase was extracted for quantification.

1 mL of this organic phase was filled into a gas chromatography vial and injected in the Thermo Scientific Trace GC Ultra (Thermo Scientific, Waltham, MA, USA), combined with a flame ionization detector (FID). After derivatization, the fatty acid methyl esters obtained from lyophilized cells were separated on a Zebron ZB-WAX column (30 m length, 0.25 mm inner diameter, 0.25 μm film thickness, Phenomenex, Torrance, USA). The split ratio was set to 1:10, and the injection volume was 1 μL. The column oven temperature was kept constant for 5 min at 120 °C and then increased to 180 °C for 20 min. The temperature was then kept constant for 10 min and further increased to 250 °C for 11 min followed by a 2 min hold. The temperature of the FID was set to 290 °C. C4 to C24 even carbon-saturated fatty acid methyl esters (FAMEs) were used for quantification and peak identification.

### Quantification of PET monomers using High-Performance Liquid Chromatography (HPLC)

Ethylene glycol measurements were conducted through HPLC-WVD-RI using an UltiMate 3000 HPLC system. This system comprised the TCC-3000SD column compartment, a WPS-3000SL autosampler, an ISO-3100SD pump, a WVD-3100 variable Wavelength Detector set at 210nm, and the SHODEX RI-101 refractive index detector sourced from Showa Denko Europe GmbH in Munich, Germany. Ethylene glycol (EG) elution was accomplished using a Metab-ACC ion exchange column with dimensions of 300 × 7.8 mm and a particle size of 10 μm, procured from ISERA in Düren, Germany. In the isocratic method, a mobile phase of 5 mM H_2_SO_4_ was employed, with a consistent flow rate of 0.6 mL min^−1^. The column oven temperature was maintained at 60 °C. Each injection contained a volume of 5 μl.

A set of standards was utilized to calibrate EG concentrations, including concentrations of 1.25 mM, 2.5 mM, 5 mM, 10 mM, 20 mM, and 40 mM.

Terephthalic acid was quantified through HPLC using the UltiMate 3000 HPLC system. This system was comprised of the TCC-3000SD column, a WPS-3000TSL analytical autosampler, an HPG-3400SD pump, and the MWD-3000 Multiple Wavelength Detector, which was set to wavelengths of 254 nm and 280 nm for detection. Terephthalic acid was eluted using an ISApher 100–5 C18 BDS gravity column (250 × 4.0 mm, particle size 5 μm; ISERA, Düren, Germany). The elution process employed a binary gradient comprising 90% formic acid (0.1%, v/v, in ultrapure water) and 10% acetonitrile (ACN). From the 2nd to the 14th minute, the gradient linearly transitioned from 90% formic acid and 10% ACN to 100% ACN, and this composition was maintained for an additional 2 min. Returning to the initial state, the period from the 16th to the 18th minute saw the restoration of 10% ACN and 90% formic acid, maintained until the measurement's end.

A sample injection volume of 1 μl was utilized, while the flow rate was set at 0.8 mL min^−1^. The column oven temperature was maintained at 40°C. Calibration of TA relied on a series of standards with concentrations ranging from 0.5 mM to 40 mM, including concentrations of 1.25 mM, 2.5 mM, 5 mM, 10 mM, 20 mM, and 40 mM.

## Data Availability

All data generated in the course of this study has been incorporated into the published article.
